# PAYROLL-BASED STAFFING MEASURES FOR NURSING HOMES

**DOI:** 10.1093/geroni/igz038.241

**Published:** 2019-11-08

**Authors:** Christianna Williams, Qing Zheng, Alan White

**Affiliations:** Abt Associates, Inc., Durham, North Carolina, United States

## Abstract

The Centers for Medicare & Medicaid Services (CMS) developed the Payroll-Based Journal (PBJ) system for nursing homes to electronically submit direct care staffing information based on payroll and other auditable data. In spring 2018, CMS started reporting PBJ-based staffing measures on Nursing Home Compare. The objective of this research is to examine nursing home staffing patterns using PBJ data. We created measures of staffing hours per resident day, using PBJ staffing information and resident census calculated from MDS assessments. We examined how PBJ staffing levels varied for different types of nursing homes and the relationship between staffing and performance on other parts of CMS’s Five-Star Quality Rating System. We also examined weekday/weekend variation in staffing levels. We tracked about 15,650 nursing homes from 2017 to 2018. The average staffing level was 3.85 hours per resident day, of which 0.66 hours were for RNs. Average staffing levels were higher for smaller, non-profit, and hospital-based facilities. They were also higher for facilities with higher health inspection and quality measure ratings. Staffing levels were about 17% lower on weekends than on weekdays, and RN staffing was 38% lower on weekends. About 20% of facilities had one or more weekend day without any RN staffing in the quarter, while only 8% of facilities had any weekday without RN staffing. The use of payroll-based staffing measures improves the accuracy of the staffing information reported on Nursing Home Compare, providing consumers with additional quality-related information that can help guide their nursing home placement decisions.

